# An Adjustable Pneumatic Planter with Reduced Source Vibration for Better Precision in Field Seeding

**DOI:** 10.3390/s24113399

**Published:** 2024-05-25

**Authors:** Jyotirmay Mahapatra, Prem Shanker Tiwari, Krishna Pratap Singh, Balaji Murhari Nandede, Ramesh K. Sahni, Vikas Pagare, Jagjeet Singh, D. J. Shrinivasa, Sandip Mandal

**Affiliations:** 1 Division of Agricultural Engineering, Indian Agricultural Research Institute, Pusa, New Delhi 110012, India; pagare.vikas7991@gmail.com (V.P.); jgskgp@gmail.com (J.S.); 2 ICAR-Central Institute of Agricultural Engineering, Nabi Bagh, Bhopal 462038, Madhya Pradesh, India; pst@ciae.res.in (P.S.T.); krishnapratap.singh@icar.org.in (K.P.S.); nandede.murhari@icar.gov.in (B.M.N.); smandal2604@gmail.com (S.M.); 3 Center for Precision and Automated Agricultural Systems, Washington State University, Prosser, WA 99350, USA; 4 Department of Agricultural Engineering, Institute of Agricultural Sciences, Banaras Hindu University, Varanasi 221005, Uttar Pradesh, India; shrinivasadj@bhu.ac.in

**Keywords:** planter vibration, electronic control, cotton seed, precision planting, pneumatic planter

## Abstract

The growing demand for agricultural output and limited resources encourage precision applications to generate higher-order output by utilizing minimal inputs of seed, fertilizer, land, and water. An electronically operated planter was developed, considering problems like ground-wheel skidding, field vibration, and the lack of ease in field adjustments of ground-wheel-driven seed-metering plates. The seed-metering plate of each unit of the developed planter is individually driven by a brushless direct current (BLDC) motor, and a BLDC motor-based aspirator is attached for pneumatic suction of seeds. The revolutions per minute (RPM) of the seed-metering plate are controlled by a microcontroller as per the received data relating to RPM from the ground wheel and the current RPM of the seed-metering plate. A feedback loop with proportional integral derivative (PID) control is responsible for reducing the error. Additionally, each row unit is attached to a parallelogram-based depth control system that can provide depth between 0 and 100 mm. The suction pressure in each unit is regulated as per seed type using the RPM control knob of an individual BLDC motor-based aspirator. The row-to-row spacing can be changed from 350 mm to any desired spacing. The cotton variety selected for the study was RCH 659, and the crucial parameters like orifice size, vacuum pressure, and forward speed were optimized in the laboratory with the adoption of a central composite rotatable design. An orifice diameter of 2.947 mm with vacuum pressure of 3.961 kPa and forward speed of 4.261 km/h was found optimal. A quality feed index of 93% with a precision index of 8.01% was observed from laboratory tests under optimized conditions. Quality feed index and precision index values of 88.8 and 12.75%, respectively, were obtained from field tests under optimized conditions.

## 1. Introduction

Precision in performance is important when developing seeding equipment. Different types of planters are available, based on the power source, type of seed-metering system, seed-metering plate drive system, and previous land preparation. In general, horizontal, vertical, inclined plate, brush, finger pick-up, and belt-type seed-metering systems are available for mechanical metering of seeds. However, precision planting and lower seed damage in pneumatic planters have made these more popular. A pneumatic seed-metering system is more advantageous than conventional seed metering. Better seed singulation and quality feed index with lower standard deviation in seed-to-seed spacing are encouraged in pneumatic planting. Delicate handling of seed in a pneumatic planter reduces mechanical damage to seed. Pneumatic planters can be classified as positive-pressure pneumatic planters or suction-type pneumatic planters. Again, some suction-type planters are assisted with seed knockout devices or positive pressure for reliable, timely release of seeds. Planters with cylindrical seed metering or plate-type seed metering are common. Higher pressure requirements and pressure variation across rows in cylindrical seed metering encourage the use of plate-type seed metering. Worldwide, many famous agricultural machinery manufacturing industries like John Deere, Horsch, Maschio, and Kinze adopt plate-type pneumatic seed metering in their models [1]. Li et al. developed a double-row pneumatic metering system for Brassica chinensis [2]. The seed-metering plate of a planter may be driven by a ground-wheel drive or a forward-travel-sensing electronic motor system. Ground-wheel drive is a simple, low-cost option, but not precise enough. Skidding of ground wheels occurs due to loose soil or improper ground contact, causing low traction development. Skidding is also caused when the power requirements for the rotation of the seed-metering plate, chain, and sprocket drive exceed the developed power at the ground wheel. Seed dropping stops, leading to a higher miss index with skidding. Mechanical power transmission systems generate vibration. The vibration disturbs the planter performance. An electronic forward-speed-sensing system with a seed-metering plate driven by a DC motor can avoid such problems. The planter’s performance is affected by different machine and operational parameters. The orifice size of the seed metering plate and vacuum pressure for seed suction are two important machine parameters affecting the quality of seed sowing. Similarly, the peripheral speed of the seed-metering plate affects its performance. Optimization of these three important parameters according to seed type is essential.

Cotton is a crop of global importance due to its diversified use, worldwide cultivation covering major areas, and the large numbers of dependent industries and people they employ. Globally, 33 Mha of agricultural land is used for cotton production [3]. Countries including India, USA, China, Pakistan, Brazil, Australia, Turkey, and Uzbekistan are the major producers of cotton. The fiber of cotton has unique characteristics and provides comfort as apparel, which is not comparable with silk, jute, banana fiber, or other natural fibers. Cotton fiber has undergone increasing demand despite the introduction of synthetic fibers. Cotton seed is also a raw input for many industries. Cotton oil is used in cooking and biodiesel production [4]. The cake produced following oil extraction is used as a protein-rich feed for animals [5]. Healthy cotton seeds are carefully processed and treated in seed industries [6]. Fuzzy cotton seeds are used for paper currency production [7]. Presently, cotton occupies 10.5 Mha of agricultural land in India [8]. The area under cotton cultivation has increased substantially in the past and has scope to further increase in the future. India remains among the top cotton-producing and -exporting countries in the world [9]. The introduction of Bt cotton has played a significant role in India’s cotton production, which has multiplied from 15.8 million bales since its introduction in 2002 to reach 31.5 million bales in 2008 [10]. The pneumatic planters available are generally operated by a PTO-driven aspirator. This causes vibration in the metering system and negatively affects the performance. This implement weighs 320 kg [11]. The aspirator, with flywheel and frame, is the major contributor to the high weight, which causes a substantial increase in overall draft and power consumption. The coupling system is an extra part required for power transmission and also requires more time for setting up. The large, massive body parts make these planters complex and increase the material as well as manufacturing costs. Li et al. [12] studied the effect of an airflow distribution device on loss of air pressure and uniform distribution of airflow. A CFD simulation study was carried out to optimize the structure of the distributor to minimize pressure loss and variation in pressure.

Liu et al. [13] studied the field factors causing vibration and its effect on a no-tillage suction-type precision planter. The effect of vibration on seeding quality and different reasons for field vibrations were discussed. The sources of vibration were described as external factors: the land wheel and furrow opener, planter power transmission and working process, and the power unit. Zhai et al. [14] investigated the effect of row unit vibration on planting quality using John Deere Max Emerge and Exact Emerge planters in strip-tilled and no-tilled fields, respectively. In each case, three single-axis accelerometers were fitted in the left, right, and central planting row units of the planter. The conditioned accelerometer signal as well as ISObus and GNSS data were recorded on an SD card. These recorded data were later analyzed using CANoe and Matlab software (R2015b). The frequency of vibration was found to be in the range between 3 and 10 Hz. An increase in the frequency of vibration was not noticed with speed, but an increment in amplitude was. The standard deviation in plant spacing was observed to increase with an increase in speed. The miss index was found to increase with an increase in vertical acceleration, but the multiple index remained unaffected. Yu et al. [15] studied the effect of vibration on seeding performance of a precision-hole rice seeder and emphasized the necessity of a system to reduce vibrational impact. Hou et al. [16] developed an elastic-tooth-type straw cleaning roller for no-tillage precision planters to reduce the effect of vibration. Cujbescu et al. [17] tested precision planter vibration under different tillage conditions and speeds of operation. The field vibration was simulated under laboratory conditions. The drive wheel was in contact with an irregular surface. A rubber belt was used for this purpose. The belt surface was mounted with hemispherical shapes of 30 to 100 mm in diameter. The different sizes of hemispheres were changed until the achievement of measured field level vibrations. Increased vibration and decreased precision in planting resulted from increased forward speed and field irregularities. The field vibration had a significant negative effect on the performance of the planter.

The present research was carried out to address the shortcomings identified in previous research on planters. Looking into the above points critically and after in-depth analysis of the current cotton-cropping scenario in India, the lack of efficient precision planting equipment is clear. The present study has been planned to overcome the problems associated with existing pneumatic planters, like high source vibration, ground-wheel skidding, and complicated adjustments.

## 2. Materials and Methods

A pneumatic planter in which individual row units were independently operated by BLDC motors was developed. Each unit was comprised of two BLDC motors. One was used for driving the seed-metering plate, while the other was for the aspirator blower. The suction pressure in each row unit could be easily regulated by the RPM control knob of the aspirator. The torque required to rotate the seed-metering plate under evaluated optimum conditions was measured. A DC motor able to provide 1.5 times the required torque was selected. Considering the higher efficiency, dynamic response, speed torque characteristics, and operational life of the BLDC motor, it was found appropriate for the purpose. An Arduino Mega microcontroller board (Arduino Mega 2560, Arduino, Italy) was used as it was cheaper and open source with high processing frequency. The Arduino Mega 2560 is a microcontroller board based on the ATmega2560. It has 54 digital input/output pins (of which 15 can be used as PWM outputs), 16 analog inputs, 4 UARTs (hardware serial ports), a 16 MHz crystal oscillator, a USB connection, a power jack, an ICSP header, and a reset button. A 1000 PPR rotary incremental optical encoder (Orange, 3806-OPTI-1000-AB-OC, Shanghai, China) was used in the system for accurate measurement of the degree of rotation.

### 2.1. Process of Electronic Sensing and Control

The desired seed spacing can be maintained only when the RPM of the seed metering plate is regulated according to the forward speed of the tractor. A spring-loaded auxiliary ground wheel coupled to an optical incremental encoder (encoder 1) was provided in the developed equipment to sense the forward speed. The pulses generated from the encoder were read by the programmed Arduino Mega, and the RPM value was calculated. The ground wheel diameter and seed spacing were programmed to be input from the keyboard. The corresponding RPM of the seed-metering plate was calculated using these three inputs in the equation given below:

RPM of seed-metering plate = (GW RPM × π × GW diameter)/(22 × S)(1)

where GW stands for ground wheel, GW diameter is in m, and S is set seed spacing in meters.

The introduction of a feedback loop system can provide better regulation of the RPM of the seed metering plate. Therefore, another optical incremental encoder (encoder 2) was coupled to the driving shaft of one of the seed-metering plates. This encoder was used to measure the RPM of the seed-metering plate in real time and feed it to the system. Thus, the required RPM as per encoder 1 and the current RPM as per encoder 2 could be compared each time to calculate the RPM value to be increased or decreased at the seed-metering plate. This value was considered the error.

**Figure 1:**
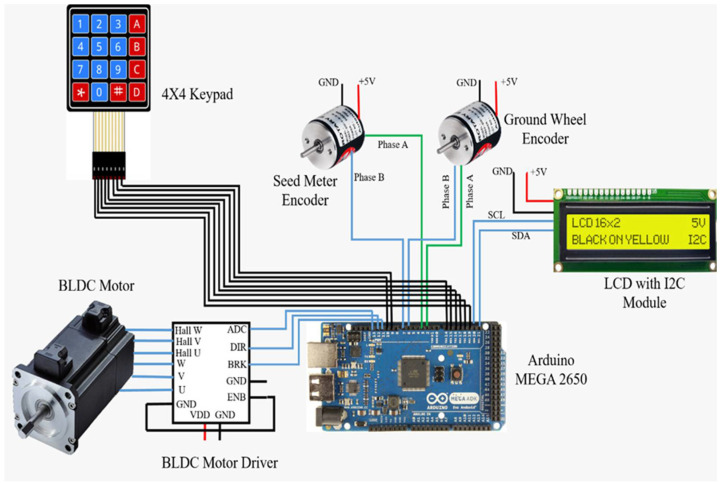
Circuit diagram representing connection to a single motor and driver.

The driving shaft of the seed-metering plate in each row unit was coupled to the output shaft of the respective BLDC motor (Rhino Motion Control Solutions, RMCS-2025, Pune, India). All four BLDC motor drivers were connected to the Arduino Mega through strip wires. The circuit diagram is presented in Figure 1. The microcontroller regulated the drivers (Rhino Motion Control Solutions, RMCS-3002, Pune, India) of the BLDC motors to supply the voltage necessary to neutralize the error. The voltage was regulated by a digital PWM signal created by the microcontroller. The PID technique was incorporated into the feedback loop to rectify the error RPM efficiently. The PWM value was regulated through PID to reach the set point within a quick response time. The Arduino Mega was programmed using the open-source Arduino IDE 1.8.19 platform to perform the above task.

### 2.2. BLDC Motor-Based Aspirator

BLDC motors are efficient and produce less noise and vibration due to their brushless design, which eliminates mechanical friction, and advanced control techniques such as PWM, optimizing performance and reducing operational disturbances. Each row unit has an individual BLDC motor-based aspirator (Wonsmart, WS9290-12-220-S200, Ningbo, China) with driver (Wonsmart, WS1230DY04V01-SRP004, Ningbo, China). This reduces vibration in comparison to a PTO-driven central mechanical aspirator. Reduced vibration improves performance. The adjustment of negative pressure according to seed type and variety becomes easy. It facilitates intercropping as negative pressure in each row can be individually set. No loss of negative pressure or variation of negative pressure across rows occurs.

### 2.3. BLDC Motor for Driving the Seed Metering Plate

The seed-metering plates are rotated by individual BLDC motor. Transmission of vibration is reduced compared with a ground-wheel-based chain-and-sprocket driving system. Reduced vibration improves performance. The desired seed-to-seed spacing can be easily achieved using the user input panel. The problem of ground-wheel skidding has been resolved. The power transmission is carried out via flexible electric cables instead of a mechanical chain and sprocket. Hence, the row units are easy to slide to set different row-to-row spacing.

### 2.4. Overview of Proposed Work

**Figure 2:**
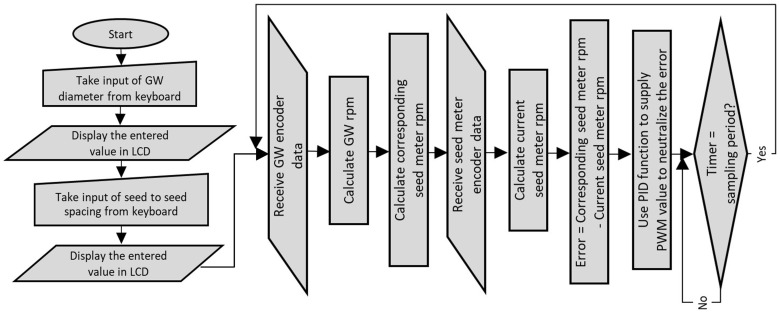
Flow diagram representing program architecture.

**Figure 3:**
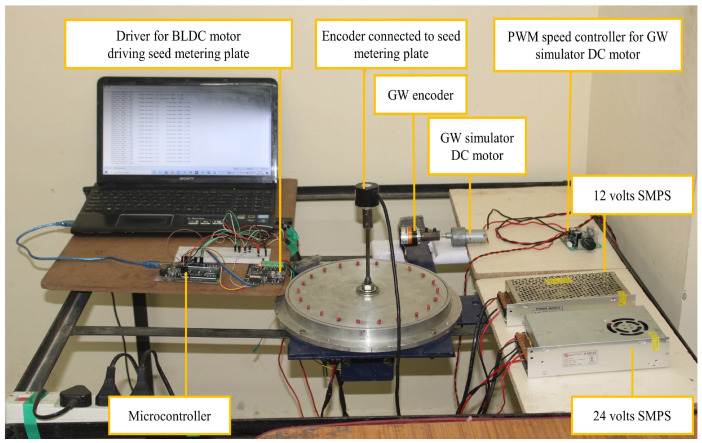
Test stand for tuning of PID controller.

The flow diagram in Figure 2 shows the flow process adopted to implement the pneumatic planter for cotton seed. Figure 3 represents the test stand for PID tuning.

Tuning the PID parameters to minimize the setting time to achieve the desired set point while also minimizing overshoot and undershoot ultimately decreased the errors in speed synchronization between the seed-metering plate and the forward speed of the tractor. Therefore, it can be deduced that precise tuning of the PID system improved the accuracy of seeding. A test stand was developed to establish the values of the best performing proportional constant (K_P_), integral constant (K_I_), and derivative constant (K_D_). A row unit was mounted on the stand and coupled to an incremental optical encoder. The BLDC motors engaged to drive the seed-metering plates were connected to the BLDC motor drivers. This driver was supplied with a 24-volt power supply from a 24-volt SMPS. The aspirator motor was connected to its driver. This driver was supplied with a 12-volt power supply from a 12-volt SMPS. The optimum vacuum pressure was supplied. The GW encoder was coupled to a 12-volt DC motor. The RPM of this DC motor was regulated through a PWM regulator, and power was supplied from the same 12-volt SMPS. The driver of the BLDC motor and both the encoders were connected to the Arduino Mega using breadboard and jumper wires. The Arduino Mega was connected to a laptop through a USB cable. The values of K_P_, K_I_, and K_D_ were changed in the program according to trial and error. In this process, the value of K**_p_** was adjusted to find a maximum value to reach the set point with minimal setting time and oscillation. Following this, the value of K**_I_** was adjusted with a gradual increment so that steady-state error could be corrected without causing instability. Finally, the value of K**_D_** was adjusted to enhance the stability of the response. The program was uploaded to the Arduino Mega after each change in value, and the corresponding response of the process variable was observed in the Arduino serial plotter. The response was observed in terms of the magnitude of overshooting, undershooting, and response time to reach the set point. Minimal overshooting and undershooting were observed with a quick setting time of 0.5 s when K_P_, K_I_, and K_D_ values were set at 1.33, 0.01, and 0.007, respectively.

### 2.5. Power Supply System

The motor driving the seed-metering plate was 24-volt DC-operated and the aspirator motor was 12-volt DC-operated. The power requirements for the aspirator motor (P_12_) and seed-metering-plate driving motor (P_24_) were separately calculated as the product of the number of rows and the power required by each individual motor (Equations (2) and (3)). The current supply required in the 12-volt system and 24-volt system was calculated by dividing the respective power requirements by the respective supply voltage (Equations (4) and (5)).

P_12_ = n × P_aspirator_ (2)

P_24_ = n × P_motor_ (3)

I_12_ = P_12_/12 (4)

I_24_ = P_24_/24 (5)

To serve this purpose, a lightweight, high-charging current and a supplying-current-capable, high-capacity, durable battery were required. The above characteristics well matched those of lithium iron phosphate batteries (LiFePO_4_). The selected battery had a capacity of 54 Ah, a supply voltage of 12 volts, and a maximum charging and discharging current of 20 amps. A supply voltage of 24 volts was generated via connecting two of these batteries in series. The power supply to each row unit had to be equal and hence, the number of batteries for the 12-volt or 24-volt supply needed to be a whole number multiple or factor of the number of row units. The total current required was divided by the supplied current of a single battery for the 12-volt and a 24-volt supply. The calculated values were 3.2 and 0.45 for the 12-volt and 24-volt systems, respectively. The nearest higher whole number to the obtained result which satisfied the condition of multiple or factor of the number of row units was calculated. Thus, for the 12-volt and 24-volt systems, 4 and 1 batteries were required, respectively.

### 2.6. Seed to Seed Spacing

**Figure 4:**
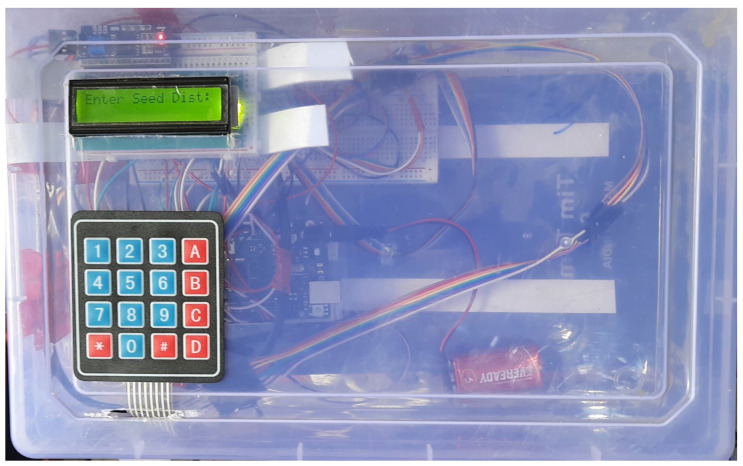
The input control panel with keyboard and display for seed-to-seed spacing.

The required seed-to-seed spacing can be provided as input to the microcontroller program through the 4 × 4 keyboard matrix (Figure 4). The input value can be viewed on the 2 × 16 LCD panel. The optimal peripheral speed of the seed metering plate can be maintained regardless of seed-to-seed spacing. This requires a change in forward speed with a change in seed-to-seed spacing. The forward speed of the tractor must be proportionally increased or decreased with the seed-to-seed spacing. The forward speed according to the set seed spacing is calculated using the equation given below:

Forward speed = Optimal RPM of seed meter × p × S × 60/1000(6)

where forward speed is in km/h, p is the number of orifices in the seed metering plate (22 in our design), and S is the set spacing in m.

### 2.7. Row to Row Spacing and Depth of Planting

**Figure 5:**
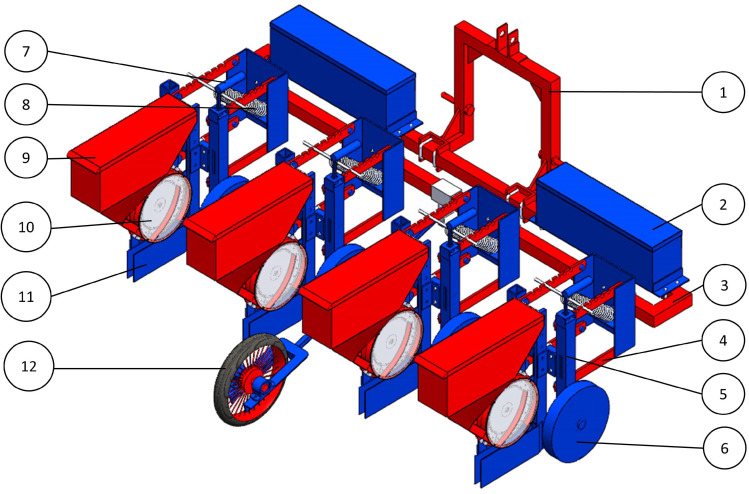
Designed electronically controlled and operated tractor-drawn four-row pneumatic cotton planter. (1: three point hitch, 2: battery box, 3: main frame, 4: parallelogram linkage, 5: H frame, 6: gauge wheel, 7: depth control handle, 8: tension spring, 9: hopper, 10: seed metering unit, 11: furrow opener, 12: auxillary ground wheel).

Cotton seeds are planted at different plant-to-plant and row-to-row spacings, and these values vary with soil type, availability of irrigation, seed variety, mechanization practices, and agroclimatic zones. The different row-to-row spacings commonly adopted are 0.30 m, 0.45 m, 0.60 m, 0.90 m, and 1.2 m [18]. The row spacing in the developed planter can easily be changed by sliding the modular row units on the main frame. Sliding these units to set a different spacing only requires loosening the U bolt. This can be tightened back after sliding the row units to the desired position. Since the seed metering system is powered by electric cables, adjusting the row spacing becomes easier. The designed planter is shown in Figure 5.

### 2.8. Depth Control Arrangement

The depth of planting is important for seed vigor [19,20]. A uniform depth of seeding is required for uniform germination and growth of plants [21]. The general planting depth recommended for cotton varies from 13 to 38 mm [22,23,24].

**Figure 6:**
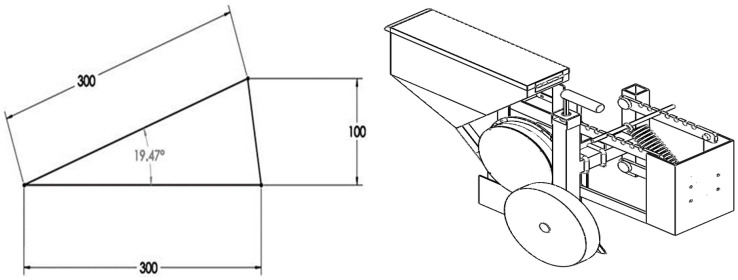
Line diagram illustrating angular movement to cover a 100 mm change in field surface height (**left**); one row unit of the planter (**right**).

**Figure 7:**
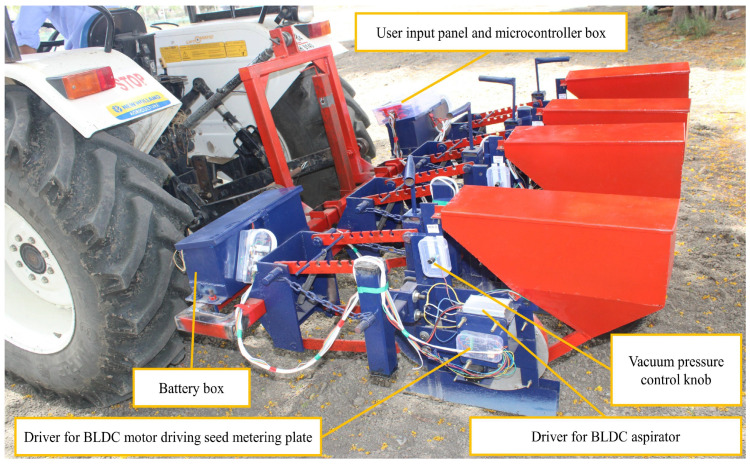
Developed electronically controlled and operated tractor-drawn four-row pneumatic cotton planter.

A parallelogram linkage system was engaged between the main frame and each row unit. A side gauge wheel performs better than a rear or front gauge wheel [21]. A depth gauge attached to the side of each seed-metering unit was provided for depth adjustment and maintenance [25]. An adjustable tension spring acts on the parallelogram linkage system to press the row unit down until the depth set at the gauge wheel is achieved. This maintains the set depth while providing adequate stability to the row unit [26]. The spring tension can be varied as per soil conditions and type by shifting its movable end. The upper two bars of the parallelogram system have been provided with seven steps to change the spring tension. A downforce higher than 600 N is required to achieve better uniformity in depth [27]; the dead weight of the hinged row unit and spring tension force must reach this value. The dead weight of the hinged part of a row unit is 382.32 N and the remaining force of 217.68 N is required to be supplied by spring tension. The spring has been designed to provide the above force when elongated and engaged with the first step. A bracket was developed to serve as a hinged support for the parallel linkages, a fixed support for one end of the spring, and a connector for the row unit to the main frame. The other end of the parallel linkage was hinged to the H-shaped frame, which holds the depth regulation system. The center-to-center distance between the hinged points of all parallel linkages was kept at 300 mm, as a lower change in angle is desired when the parallel linkages are working [25,28]. The associated maximum angle and single row unit of the planter are shown in Figure 6. The depth can be regulated by rotating the handle connected to a screwed shaft. The screwed shaft lifts or lowers the movable square box on which the gauge wheel is mounted. The movable square box fits inside the H-shaped frame that guides it. The furrow opener operates with reference to the gauge wheel and, thus, higher or lower depths can be achieved by lifting or lowering the gauge wheel, respectively. The developed planter is shown in Figure 7.

### 2.9. Performance Optimization

The seed-metering plates were optimized for the metering of cotton seed. Cotton seed variety RCH 659 was used. Important parameters like forward speed, orifice size, and vacuum pressure were optimized according to the data obtained from sticky belt runs. Central composite rotatable design was applied and 20 combinations of independent parameters were generated for experimentation. Each experiment was replicated five times. The seed spacings were recorded and later analyzed to calculate the indices, using the following equations (Equations (7)–(9)):

MII = n_1_/N (7)

MUI = n_2_/N (8)

PI = S_d_/S (9)

where MII is the miss index, MUI is the multiple index, PI is the precision index, n_1_ is the number of seed spacings more than 1.5 times the set spacing, n_2_ is the number of seed spacings less than 0.5 times the set spacing, N is the total number of the seed spacings, S_d_ is the standard deviation of the seed spacings that are excluded under the miss index and multiple index calculations, and S is the set spacing.

## 3. Results and Discussion

In this section, we report the evaluation results for our model’s characteristics including miss, multiple, and precision indices for various combinations of independent parameters. The results are tabulated in Table 1. Quadratic polynomial equations were developed through ANOVA. The effect of each independent parameter and the interaction effect of two independent parameters on each dependent parameter were calculated from the ANOVA.

Results obtained from different experimental runs are presented in Table 1, where O_d_, V_p_, and F_s_ stand for orifice diameter (mm), vacuum pressure (kPa), and forward speed (km/h), respectively. These are independent parameters, whereas miss index (%) MII, multiple index (%) MUI, and precision index (%) PI are dependent parameters.

The miss index was found to be influenced by orifice diameter, vacuum pressure, and forward speed at 1% level of significance. An increased miss index was found to be associated with decreased orifice diameter and vacuum pressure. This is because with a decrease in orifice diameter, the suction force at the orifice decreases and a sufficient area of contact is not provided by the reduced suction area. Vacuum pressure also affected the miss index in a similar manner. The reduced suction force applied to the seed failed to capture it when the vacuum pressure was low. In some cases, although the seed was sucked, the suction force was not enough to hold the seed against the reaction force generated on the seed upon striking the seed-metering plate. An increase in forward speed was found to positively affect the miss index. The decreased interaction time between the seed and seed-metering plate caused a higher miss index at increased speed. Despite the suction, the seed were unable to be carried forward as they failed to achieve the required kinetic energy within the short span of time.

An increase in orifice diameter increased suction force and orifice area. This resulted in seeds being sucked with a higher force and more than one seed being picked up, due to provision of a better area of exposure and contact with each seed. An increase in vacuum pressure also resulted in an increase in the multiple index. Increased suction force pulled more seeds, resulting in a higher multiple index. As orifice size or vacuum pressure increased, the radial distance from the center of the orifice at which the terminal velocity of air exceeded the terminal velocity of the seed increased. Thus, the increased suction zone picked up multiple seeds. A higher suction force was able to pick up seeds successfully, overcoming the reaction force generated when a seed strikes the seed-metering plate. It was also able to accelerate the picked-up seeds to gain the kinetic energy of the seed-metering plate.

**Figure 8:**
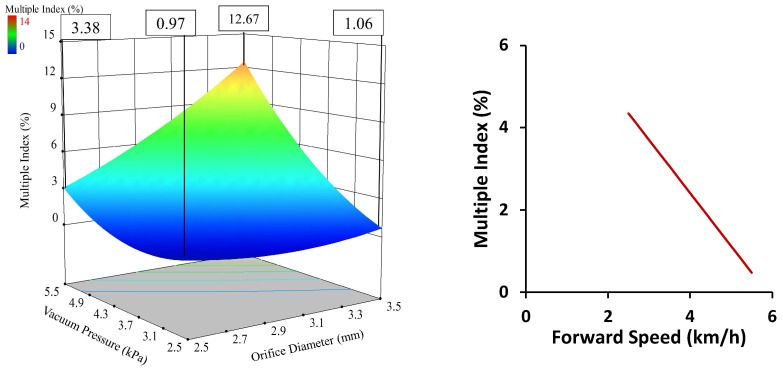
Effect of independent parameters on multiple index.

**Figure 9:**
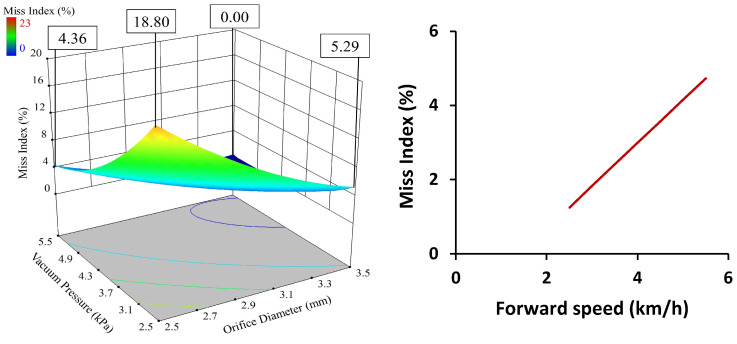
Effect of independent parameters on miss index.

An increase in forward speed caused the multiple index to decrease. The seed and seed-metering plate interaction time reduced with increased forward speed. The required acceleration of seed to obtain the kinetic energy of the seed-metering plate also increased. This occurred as the picking time reduced and the peripheral velocity of the metering plate increased. The same suction force was unable to provide the required acceleration to seeds at higher speeds, which reduced the multiple index and increased the miss index. The interaction of orifice diameter and vacuum pressure was revealed to influence the multiple index at a 1% level of significance. This interaction was expressed through the significant changes in the multiple index value when the vacuum pressure changed at a constant value of orifice diameter, or vice versa. The graph presented in Figure 8 clearly shows the phenomenon, which occurred because an increase in orifice diameter increased the multiple index by providing more suction area, greater area of contact between the seed and seed-metering plate, and also increased suction force. Similarly, increased vacuum pressure increased the multiple index through a stronger suction force and area. A change in any one parameter was able to overcome the effects of the others in a significant way. The interaction of orifice diameter and vacuum pressure was revealed to influence the miss index at a 1% level of significance. This interaction was expressed through the miss index value changing significantly when the vacuum pressure changed at a constant value of orifice diameter, or vice versa (Figure 9). This is because an increase in orifice diameter reduced the miss index by providing more suction area, increased area of contact between the seed and seed-metering plate, and greater suction force. Similarly, increased vacuum pressure reduced the miss index through increasing the suction force and area. A change in any one parameter was able to overcome the effects of the others. The multiple index was found to be affected by orifice diameter, vacuum pressure, and forward speed at a 1% level of significance.

**Figure 10:**
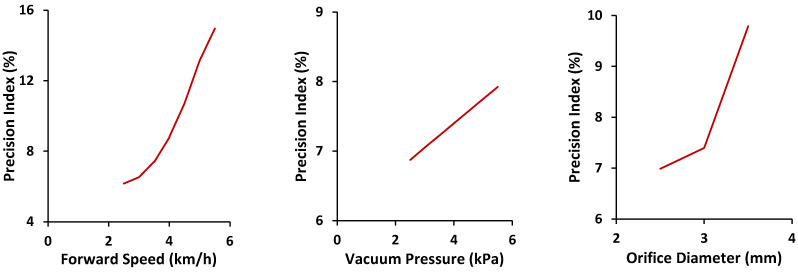
Effects of independent parameters on precision index against forward speed (**left**), vacuum pressure (**middle**), and orifice diameter (**right**).

The effects of different independent parameters on the precision index are shown in Figure 10. The precision index increased with an increase in orifice diameter. Increased vacuum pressure was also found to increase the precision index. An increased vacuum pressure created a stronger suction force, and picked-up seeds were strongly attached to orifices, creating some degree of physical engagement between the seed and the seed-metering plate. The vacuum pressure cutoff in the vacuum chamber did not seal the vacuum pressure completely. At higher levels of vacuum pressure, the pressure leakage increased. This leakage of pressure affected the fall of seeds. All seeds did not fall exactly at the cutoff, as the leaked pressure was still acting on them. Seeds started falling from different positions according to their mass after the vacuum pressure was cut off. The increase in forward speed was also found to increase the precision index. This could be due to the higher effect of inertia of motion on the seeds. At the same velocity, the inertia force obtained by seeds of different mass varied. After the vacuum pressure was cut off, the seeds travelled under the combined action of gravity and the force of inertia.

The orifice diameter and forward speed were found to affect the precision index at a 1% level of significance, while the vacuum pressure was found to affect it at a 5% level of significance. A larger orifice provided a higher degree of engagement between the seed and the seed-metering plate. This means a seed had more opportunity for a higher portion of it to be sucked into the orifice and higher peripheral contact between the seed and the orifice. At the cutoff of vacuum pressure, the seed moved under the resultant force caused by inertia of motion as well as gravity. Here, the seed did not fall immediately, because of the physical interaction with the orifice. Hence, seed spacing was affected, resulting in a higher precision index. This effect of speed on seed spacing was greater at higher speeds. Similar effects have been reported by Panning et al. [29], Yazgi and Degirmencioglu [30], and Ismail [31].

### Comparative Analysis

**Figure 11:**
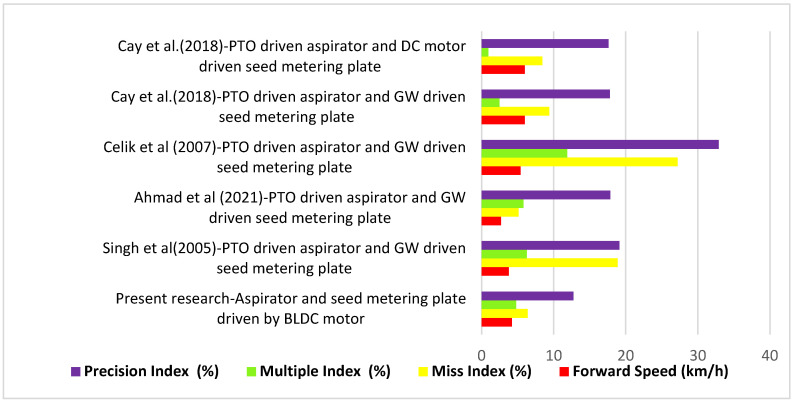
Comparative study and analysis of the results of proposed model with previous research [33,41,44,45,46] on the basis of different driving and suction systems.

The summary of comparative states of our proposed model against the existing research is presented in Figure 11. Similar relationships between orifice diameter, vacuum pressure, speed, miss index, and multiple index were reported by Barut and Ozmerzi [32], Singh et al. [33], Yazgi and Degirmencioglu [30], Yasir et al. [34], Ibrahim et al. [35], and Bhimani et al. [36]. Dixit et al. [37] evaluated the comparative performance of pneumatic planters and an inclined plate planter. They further observed that parameters like orifice diameter, vacuum pressure, and forward speed had a considerable impact on precision index.

The obtained data were used for optimization using the RSM software Design Expert. The orifice diameter and vacuum pressure were kept in range, while forward speed was maximized with low weightage. All the dependent parameters were minimized with high weightage. A miss index of 4.030%, a multiple index of 1.385%, and a precision index of 7.677% were predicted for an optimized orifice diameter of 2.947 mm, vacuum pressure of 3.961 kPa, and forward speed of 4.261 km/h. The miss index, multiple index, and precision index were found to be 5, 2, and 8.01% when evaluated under laboratory conditions with optimized parameters. Thus, a quality feed index of 93% was observed. Similar results for the optimization of cotton seed have been reported by many researchers. Singh et al. [33] obtained an optimized vacuum pressure of 2 kPa, a cone angle of 120°, and a speed of 0.42 m/s with a metering plate with an orifice size of 2.5 mm for cotton seed. A quality feed index of 94.7% with an 8.6% coefficient of variation in spacing was obtained using the above optimal conditions in their study. An optimized orifice diameter of 3 mm and vacuum pressure of 5.5 kPa were concluded by Yazgi and Degirmencioglu [30] but no optimal speed was reported, as they observed decreased performance with increased speed. A quality feed index of 99.3% was observed when the seed-metering plate was operated at a peripheral speed of 0.1 m/s, corresponding to a forward speed of 1 m/s. A peripheral speed of 0.34 m/s, vacuum pressure of 6.3 kPa, and orifice diameter of 3.5 mm resulted in a quality feed index of 90.87% and a precision index of 23.72% when tested with a seed-metering plate with 26 holes, using the Deltapine 388 variety of cotton seed [38].

The developed planter with optimized parameters was operated in the experimental fields of Central Institute of Agricultural Engineering, Bhopal, India (23.3099° N, 77.4031° E). The soil type was black cotton soil (tilled) with a bulk density of 1254 kg/m^3^. Quality feed index and precision index values of 88.8 and 12.75% were obtained from field tests. The miss index and multiple index were 6.4 and 4.8%, respectively. The average and standard deviation of seed spacing were found to be 153.22 ± 18.28, 156.73 ± 19.63, 150.71 ± 18.31, 150.82 ± 19.71, and 159.79 ± 19.67 mm in different field replications. The marginal difference between actual seed sown within a marked distance of 7.5 m was two to three seeds less than expected in different replications. Although the machine was designed for precise dropping, inaccurate inputs through the user input panel can cause more/less seeding. However, in the study conducted, the difference was primarily due to combined effect of miss, multiple, and precision index. The variation in seed weight and shape influencing the seed dropping trajectory may be attributed for precision. Although the metering plate was optimized still the variation in seed properties cause minimum multiple picking or seed missing. The field level quality feed index reported by Singh et al. [33] was 74.83% with a precision index of 19.12%. A higher quality feed index and better precision found in the present study may be attributed to reduced source vibration and skidding effects. The effects of planter vibration on performance have also been reported by Liu et al. [13], Zhai et al. [14], Yu et al. [15], Hou et al. [16] and Cujbescu et al. [17]. The driving unit, power source, and ground wheel are some of the prime factors producing vibration. The metering plates of the developed planter are rotated by individual BLDC motors, and suction is produced by individual BLDC motor-based aspirators, leading to reduced vibration. The problem of missing caused by ground wheel skidding was avoided by providing an auxiliary ground wheel to sense the forward speed and accordingly controlling the speed of the seed metering plate driving motor through PWM of the supply voltage. Feedback control along with PID was applied for real time sensing and actuation. Better performance in planting was also achieved by Jafari et al. [39], Kamgar et al. [40], Cay et al. [41], Strasser et al. [42], and Coelho [43] by employing electronic drive systems.

The obtained results were compared with the results of previous studies to show the performance of our research work. Ahmad et al. [45] conducted an experiment using different levels of tilled land condition and speeds of operation. The maximum miss index was observed when operated with higher speed and lower pulverization. The maximum multiple indices were observed when operated with a lower speed and lower pulverization. The pulverization condition of soil indirectly indicates the effect of vibration; lower pulverization shows a higher effect of vibration, adversely affecting the performance of the pneumatic planter. The quality feed index increased from 60.56 to 89.03%, and the precision index improved from 31.07 to 17.85% as the level of land preparation improved. Cay et al. [41] developed an electro–mechanic drive system for a planter and evaluated its performance under laboratory as well as field conditions. The effects of the classic drive system and the electro–mechanic drive system on the performance of pneumatic seed metering were compared. A 2.5% increase in quality feed index and a 0.18% decrease in precision index were seen under field conditions when operated with the developed electro–mechanic drive system. Celik et al. [46] studied the emergence and distribution uniformity of sunflower seed by using four different types of planters. A quality feed index of 60.90% and a precision index of 32.90% were observed with the vacuum-type pneumatic planter.

Singh et al. [33] evaluated the lab and field performance of a pneumatic planter operated under optimized conditions. The miss index, multiple index and precision index were found to increase from 1.33 to 18.88%, 4 to 6.29%, and 8.55 to 19.12% when the planter was moved from laboratory to field conditions. A quality feed index of 74.83% and a precision index of 19.12% were reported for the field distribution of seeds. A significant difference between laboratory and field performance was reported.

## 4. Conclusions

The miss index and multiple index were majorly affected by orifice diameter, vacuum pressure, forward speed, and the interaction of orifice diameter and vacuum pressure (*p* ≤ 0.01). The precision index was majorly affected by orifice diameter (*p* ≤ 0.01), vacuum pressure (*p* ≤ 0.05), and forward speed (*p* ≤ 0.01). The increase in orifice diameter and vacuum pressure increased the multiple index and decreased the miss index. The effect of an increase in orifice diameter can be minimized by decreasing vacuum pressure, and vice versa. Similarly, the effect of a decrease in orifice diameter can be minimized by increasing vacuum pressure, and vice versa. The increase in forward speed decreased the multiple index and increased the miss index. An increased precision index was noticed with increases in forward speed, orifice diameter, and vacuum pressure. Minimum precision index can be obtained with minimum orifice diameter and vacuum pressure but, on the other hand, this increases the miss index. In this way, optimization for the minimum precision index negatively affected the quality feed index. The increased variation in seed spacing was caused by increased orifice diameter and vacuum pressure that increased the degree of physical engagement between the seed and orifice. The addition of a positive seed dropping mechanism will aid in reducing the precision index without compromising the quality feed index.

Individual BLDC motors were used to replace mechanical systems for easy adjustment and reduced vibration. The comparative performance of pneumatic planters with different types of aspirators and seed-metering drives shows the enhanced performance of the developed model. A quality feed index of 93% and precision index of 8.01% were achieved in the laboratory, using an orifice diameter of 2.947 mm, vacuum pressure of 3.961 kPa, and forward speed of 4.261 km/h. The quality feed index and precision index values obtained in the field were 88.8 and 12.75%, respectively. The lower difference between laboratory and field performance was achieved through reduced source vibration through adopting an independent BLDC motor-based aspirator and seed-metering-plate driving system for each unit. This also provided better flexibility in the adjustment of vacuum pressure, seed spacing, depth, and row spacing, and produced less vibration. The demand for such electrically powered and easily adjustable farm equipment will rise with newly evolving electric tractors and solar-powered or hybrid agricultural vehicles. In large-scale manufacture, electronic circuit components can be substituted with printed circuit boards to provide dependable and resilient functionality. Retaining a redundant printed circuit board is cost-effective and can enhance reliability.

## Figures and Tables

**Table 1:** Results obtained from different experimental runs.

Sl No.	Independent Parameters			Dependent Parameters		
	O_d_	V_p_	F_s_	MII	MUI	PI
1	3.00	4.00	1.48	1	8	7.04
2	3.00	4.00	4.00	3	2	7.22
3	2.50	5.50	2.50	3	5	7.18
4	3.00	4.00	4.00	3	2	7.24
5	3.50	5.50	5.50	1	10	16.16
6	3.00	4.00	4.00	3	2	7.20
7	3.84	4.00	4.00	1	9	12.74
8	3.00	4.00	4.00	3	2	7.18
9	2.16	4.00	4.00	17	0	7.21
10	3.50	2.50	2.50	2	2	8.53
11	3.00	6.52	4.00	1	14	9.10
12	3.00	1.48	4.00	23	0	7.07
13	2.50	5.50	5.50	6	1	13.05
14	3.00	4.00	4.00	3	2	6.98
15	3.50	2.50	5.50	5	0	14.78
16	2.50	2.50	5.50	20	0	11.93
17	3.50	5.50	2.50	0	14	9.52
18	3.00	4.00	6.52	8	0	20.55
19	3.00	4.00	4.00	3	2	7.20
20	2.50	2.50	2.50	15	3	6.81

## Data Availability

The analyzed datasets are available from the corresponding author on reasonable request.

## Abbreviations

Ah	Ampere hours
ANOVA	Analysis of variance
BLDC	Brushless direct current
Bt	*Bacillus thuringiensis*
CAN	Controller area network
CFD	Computational fluid dynamics
DC	Direct current
F_s_	Forward speed
GNSS	Global navigation satellite system
GW	Ground wheel
Hz	Hertz
I_12_	Current requirement for 12-volt system
I_24_	Current requirement for 24-volt system
IDE	Integrated development environment
K_D_	Derivative constant
K_I_	Integral constant
km/h	Kilometres per hour
K_p_	Proportional constant
kPa	Kilo Pascal
LCD	Liquid crystal display
LiFePO_4_	Lithium iron phosphate
m/s	Meters per second
m	Meter
Mha	Million hectares
MII	Miss index
mm	Millimetre
MUI	Multiple index
n	Number of row units
N	Total number of the seed spacings
n_1_	Number of seed spacings more than 1.5 times the set spacing
n_2_	Number of seed spacings less than 0.5 times the set spacing
O_d_	Orifice diameter
p	Number of orifices in seed-metering plate
P_12_	Power requirement for 12-volt system
P_24_	Power requirement for 24-volt system
P_aspirator_	Power requirement of a single aspirator
P_motor_	Power requirement of a single seed metering plate driving motor
PI	Precision index
PID	Proportional integral derivative
PPR	Pulses per revolution
PTO	Power take-off
PWM	Pulse width modulation
RPM	Revolutions per minute
S	Set seed spacing
SD card	Secure digital card
S_d_	Standard deviation of the seed spacings that are excluded under the miss index and multiple index calculations
SMPS	Switch mode power supply
USB	Universal serial bus
V_p_	Vacuum pressure
